# The implication of serum HLA-G in angiogenesis of multiple myeloma

**DOI:** 10.1186/s10020-024-00860-5

**Published:** 2024-06-14

**Authors:** Chi Wang, Nai-Wen Su, Kate Hsu, Chen-Wei Kao, Ming-Chih Chang, Yi-Fang Chang, Ken-Hong Lim, Yi-Hao Chiang, Yu-Cheng Chang, Meng-Ta Sung, Hsueh-Hsia Wu, Caleb G. Chen

**Affiliations:** 1 Department of Laboratory Medicine, MacKay Memorial Hospital, New Taipei, 25160 Taiwan; 2 Department of Hematology, MacKay Memorial Hospital, Taipei, 10449 Taiwan; 3 Nursing, and Management, MacKay Junior College of Medicine, New Taipei, 25245 Taiwan; 4 Institute of Biomedical Sciences, MacKay Medical College, New Taipei City, 25245 Taiwan; 5 Department of Medical Research, Mackay Memorial Hospital, New Taipei City, 25160 Taiwan; 6 Department of Hematology, GCRC Laboratory, Mackay Memorial Hospital, New Taipei City, 25160 Taiwan; 7 Department of Medicine, MacKay Medical College, New Taipei City, 25245 Taiwan; 8 Medical Laboratory Science and Biotechnology, Taipei Medical University, Taipei, 110 Taiwan; 9 Institute of Molecular Medicine, National Tsing-Hua University, Hsin-Chu, Taiwan

**Keywords:** Multiple myeloma, Angiogenesis, Human leukocyte antigen G, Vascular endothelial growth factor, Interleukin 6, Hypoxia-inducible factor-1α

## Abstract

**Background:**

Despite the advances of therapies, multiple myeloma (MM) remains an incurable hematological cancer that most patients experience relapse. Tumor angiogenesis is strongly correlated with cancer relapse. Human leukocyte antigen G (HLA-G) has been known as a molecule to suppress angiogenesis. We aimed to investigate whether soluble HLA-G (sHLA-G) was involved in the relapse of MM.

**Methods:**

We first investigated the dynamics of serum sHLA-G, vascular endothelial growth factor (VEGF) and interleukin 6 (IL-6) in 57 successfully treated MM patients undergoing remission and relapse. The interactions among these angiogenesis-related targets (sHLA-G, VEGF and IL-6) were examined in vitro. Their expression at different oxygen concentrations was investigated using a xenograft animal model by intra-bone marrow and skin grafts with myeloma cells.

**Results:**

We found that HLA-G protein degradation augmented angiogenesis. Soluble HLA-G directly inhibited vasculature formation in vitro. Mechanistically, HLA-G expression was regulated by hypoxia-inducible factor-1α (HIF-1α) in MM cells under hypoxia. We thus developed two mouse models of myeloma xenografts in intra-bone marrow (BM) and underneath the skin, and found a strong correlation between HLA-G and HIF-1α expressions in hypoxic BM, but not in oxygenated tissues. Yet when stimulated with IL-6, both HLA-G and HIF-1α could be targeted to ubiquitin-mediated degradation via PARKIN.

**Conclusion:**

These results highlight the importance of sHLA-G in angiogenesis at different phases of multiple myeloma. The experimental evidence that sHLA-G as an angiogenesis suppressor in MM may be useful for future development of novel therapies to prevent relapse.

**Supplementary Information:**

The online version contains supplementary material available at 10.1186/s10020-024-00860-5.

## Introduction

Survival of multiple myeloma (MM) patients has been dramatically improved over the past decades with new drugs and intervention (Palumbo and Anderson 2011; Kumar et al. 2014; Pawlyn and Davies 2019). However, MM remains an incurable disease and most patients eventually relapse. One of the culprits is the interaction between myeloma cells and the bone marrow (BM) environment, which contains BM stromal cells (Vacca and Ribatti 2006), inflammatory cells (Coussens and Werb 2002), and endothelial cells (Ria et al. 2004; Zhang et al. 2005), and favors tumor growth and drug resistance (Zhang et al. 2005; Vacca and Ribatti 2006). Endothelial cells could form plentiful new blood vessels for tumor expansion or angiogenesis, which is one of the hallmarks of cancer (Hanahan and Weinberg 2011). Myeloma cell interaction with the BM microenvironment (BMM) cells could stimulate production of angiogenic cytokines that promote neovascularization (Vacca and Ribatti 2006). Thus, highly proliferating myeloma cells possess an angiogenic capability to support their growth.

Physiologically, BMM is a hypoxic niche (Spencer et al. 2014). Hypoxia elevates expression of hypoxia-inducible factor-1 (HIF-1) (Martin et al. 2011). HIF-1 and HIF-2 as the master regulators of oxygen homeostasis promote angiogenesis in various cancers (Zhong et al. 1999; Talks et al. 2000; Martin et al. 2010). One of the crucial mediators in vascular formation is vascular endothelial growth factor (VEGF), a known target of HIF-1 (Ravi et al. 2000). Angiogenesis is characteristic of cancer progression (Vacca and Ribatti 2006) and poor overall survival of MM (Jakob et al. 2006). From a murine model of MM, angiogenesis is triggered by VEGF production in the plasma cells (Asosingh et al. 2004). VEGF subsequently activates BM stromal cells, which express high levels of VEGF receptor-1 and VEGF receptor-2 that can stimulate secretion of interleukin 6 (IL-6) (Podar et al. 2001). IL-6 is a proliferative factor for MM and creates an optimal BMM for tumor expansion (Harmer et al. 2018).

Even though angiogenesis is a hallmark of cancer development, some endogenous proteins, such as thrombospondin (Dawson et al. 1997), endostatin (Boehm et al. 1997), and soluble human leukocyte antigen-G (sHLA-G) (Fons et al. 2006), could counteract microvessel formation. It has been shown that HLA-G plays a role in tumor progression and metastasis (Yie et al. 2007; Li et al. 2012; Wastowski et al. 2013). High expression of HLA-G is observed in immunosuppressive cancer patients with poor prognosis (Krijgsman et al. 2020). High levels of serum sHLA-G have also been shown to suppress immune activities in MM patients (Chumbley et al. 1994; Leleu et al. 2005; Wlasiuk et al. 2012). On the other hand, HLA-G is not a prognostic factor of survival for other types of cancers (Spurny et al. 2018; Marletta et al. 2021). HLA-G alone cannot serve as a prognostic factor for MM survival (Leleu et al. 2005). These discrepancies in clinical observations point to that the roles of HLA-G in tumor biology may be diverse and complicated. One of distinct properties of sHLA-G is its ability to inhibit endothelial cell proliferation, migration and tubule formation during vascular remodeling (Le Bouteiller et al. 2007). This proposed mechanism probably contributes to retardation of tumor growth.

The expression of HLA-G is modulated by HIF-1 at the transcriptional and translational levels in hypoxic condition (Garziera et al. 2017). Since sHLA-G also contributes to anti-angiogenesis by inducing apoptosis of endothelial cells (Fons et al. 2006), we therefore speculated that the angiogenesis switch in MM might require HIF-1-mediated regulation of VEGF and sHLA-G, particularly under hypoxia. IL-6 might also affect sHLA-G expression and support formation of new vasculature in myeloma.

In the present study, we observed that sHLA-G levels in MM patients decreased upon cancer progression. To explore it mechanistically, we established a HIF-1α-knockout (KO) cell line established by CRISPR/Cas9, and found that HLA-G expression in human myeloma cell lines under hypoxia was governed by HIF-1α. When stimulated with IL-6, HIF-1α and HLA-G in the myeloma cell lines were subjected to ubiquitination and degradation. Co-culture of endothelial HUVEC and myeloma cells stimulated formation of new tubes from endothelial cells, when the cultured medium was supplemented with IL-6 or was depleted of HLA-G. These findings provide an explanation for the clinical observation in MM patients that their sHLA-G levels were inversely correlated with serum IL-6 levels and their sHLA-G levels rebounded upon remission.

## Materials and methods

### Patients and samples

This study was approved by the Mackay Memorial Hospital (MMH) Institutional Review Board (19MMHIS346e), and was carried out in accordance with the principles of the Declaration of Helsinki. The demographic data including age and gender, and clinical data including stage, M-component isotype, estimated glomerular filtration rate (eGFR), and outcome, were listed in Supplementary Table 1. Fifty-seven patient subjects were diagnosed with relapsed myeloma between 2018 and 2021 and treated using the criteria set by the International Myeloma Working Group (IMWG) (Rajkumar et al. 2014) and the International Staging System (ISS) (Greipp et al. 2005; Rajkumar et al. 2011). In accordance with the recommendation from IMWG in 2015 (Dimopoulos et al. 2015), patients with symptomatic relapse were enrolled after first remission responding to the upfront treatments. The stage of imminent relapse (IR) was arbitrarily defined as 3 months before the symptomatic relapse. Multiple serum samples were collected and cryopreserved from patients after the frontline bortezomib-based chemotherapy every 2–3 months. Serum-free light chain (FLC) was measured to evaluate disease states: complete response (CR), very good partial response (VGPR), and partial response (PR) (Rajkumar et al. 2011). The serum FLC assay (Freelite, UK) measures absolute values of free κ and λ light chains and is reported as the κ/λ ratio (Katzmann et al. 2002). Cancer treatment responses and disease progression (i.e., distinct remission phases) were assessed using a validated computerized algorithm following the criteria set by the International Myeloma Working Group (Durie et al. 2006; Rajkumar et al. 2014).

### Cell lines and cell culture

RPMI8226, U226B1, MM.1s, and human umbilical vein endothelial cell (HUVEC) cell lines were purchased from ATCC. All MM cells were cultured using RPMI-1640 media and incubated in a humidified atmosphere (normoxia) or in 3% O_2_ (hypoxia) mimicking the physiological condition of BM (Spencer et al. 2014) at 37°C. According to the report by Spencer et al. (Spencer et al. 2014), P_O2_ in the intravascular space of bone marrow is 21.9 mmHg or 2.9%, and in the extravascular space is 13.5 mmHg or 1.8%. Because we observed that the expression levels of HIF-1α and HLA-G were similar in myeloma cells incubated in 1%-3% of P_O2_ but lower in 5% of P_O2_ (Fig S1). We arbitrarily chose 3% P_O2_ as the hypoxia setting in this study. For *HIF-1α* gene knockout (KO), the CRISP/Cas9 system using the vector (pCas-Guide-EF1a-GFP, OriGene Technologies) was employed to generate stable *HIF-1αKO* cell lines. Cells were transfected using Lipofectamine CRISPRMAX Cas9, medium was changed after 24 h. sgRNAs were designed as target sequences: 5’-TTCTTTACTTCGCCGAGATC-3’ and 5’-TGTGAGTTCGCATCTTGATA-3’. The protein levels of HIF-2α under hypoxia were not affected in *HIF-1αKO* myeloma cells (Fig S2). HUVEC cells were cultured in EGM medium (PromoCell) containing 5% FBS and maintained in an incubator with 5% CO_2_ at 37 °C.

### Immunoblotting and immunoprecipitation

Cells were lysed in RIPA buffer, and whole-cell extracts were quantified by the Bradford assay (Bio-Rad). For assessment of HIF-1α and HLA-G expression, total cell lysates were analyzed by SDS-PAGE. Immunoprecipitation was performed for 3 h at 4 °C with different antibodies including anti-ubiquitin (Santa Cruz), anti- HIF-1α (Abcam), and anti-HLA-G (Novus), which were bound to protein A–Sepharose (GE Healthcare Bio-Sciences AB). The precipitated protein samples were resolved by SDS/PAGE and transferred to PVDF membranes (Millipore). The membranes were then incubated with the indicated primary antibodies, followed by an HRP-conjugated secondary antibody. The immunoreactive bands were detected using the Western Lighting Plus-ECL system (PerkinElmer) or the Opti-ECL HRP reagent kit (Bioman).

### Determination of serum sHLA-G and cytokines

Patients’ sera were harvested at different time points and cryopreserved for determination of sHLA-G (Elabscience), VEGF (R&D), and IL-6 (R&D) levels. Their concentrations were measured using enzyme-linked immunosorbent assay (ELISA) kits according to the manufacturer’s instructions.

### In vitro capillary tube formation assay

For angiogenesis studies, µ-slides (ibidi GmbH, Germany) were coated with 10 µL per well of growth factor reduced Matrigel (including βFGF; BD Biosciences), and incubated at 37℃ for 30 min. HUVECs were plated at a density of 1 × 10^4^ cells per well. HUVECs were cultured in 50 µL of EGM/2% FBS or MM-conditioned media. For preparation of the MM conditioned media, MM cells were grown in normoxia or hypoxia (3% O_2_) in EGM/2% FBS for 24 h. The conditioned media (CM) was collected and centrifuged at 2300 × g for 5 min, and filtered through a 0.22 μm filter for subsequent experiments. To deplete VEGF or sHLA-G in the conditioned media, the media were mixed with 5 µg/mL of anti-VEGF monoclonal antibody (Bevacizumab, Roche) or anti-sHLA-G monoclonal antibody (E333, R&D) for 1 h at room temperature. For some experiments, the preparation of the MM-conditioned media was preceded by adding 10 ng/mL of IL-6 in RPMI 1640 medium (Life Technologies). According to the previous report (Lee et al. 2009), tube formation was quantified by measuring tube length and tube formation structures in the polygonal networks in each well. The total length of the tubes was calculated by summing the length of individual branches including several tube-like structures that were merged together or branched. The results were represented as the total tube length (pixels) or the average of 6 random photographic fields per experimental condition (magnification 50; Axiovert 200; Carl Zeiss MicroImaging).

### In vivo model of human MM

All procedures involving animals were performed in accordance with the recommendations for the proper care and use of laboratory animals (MacKay Memorial Hospital IACUC registration: MMH-A-S-110-022-R-R-R). The NOD.Cg-Prkdc^scid^Il2rg^tm1wjl^/YckNarl (ASID) mice deficient of the Il-2 receptor gamma chain (NARLabs, Taipei, Taiwan) were housed under specific pathogen-free conditions. We established *in-vivo* models of human MM by (1) orthotopic xenografts to the bone marrow, and (2) subcutaneous implantation, according to a previous report (Schueler et al. 2013). Specifically, twenty microliters of 1 × 10^6^ RPMI8226 cells or IL-6-transfected RPMI8226 cells in a microsyringe was injected through the joint surface of the tibia and the patellar tendon into the BM cavity of an ASID mouse. In the second *in-vivo* model of human MM by subcutaneous implantation, 1 × 10^6^ RPMI8226 cells were injected into the bone marrow cavity of a fresh tibia that was just removed from a sacrificed ASID mouse, and this hollow bone filled with RPMI8226 cells was subcutaneously implanted in another ASID mouse. After 35 days of the graft, the tibia was removed and fixed in the B5 solution for sectioning and then staining with H-E, anti-CD138 (clone B-A38, BioSB), anti-HIF-1α (clone EP1215Y, Abcam), and anti-HLA-G (clone E8N9C, Cell Signaling Technology). Mayer’s hematoxylin was used to label nuclei.

### Statistical analysis

To compare the means of serum molecules at different disease phases from patients regularly followed up, paired *t*-test was used. The sample distribution of numerical variables was determined by the Kolmogorov-Smirnov test before using Pearson’s correlation coefficients to evaluate the degree of linear correlation. The results were shown as median if not otherwise mentioned. The threshold for statistical significance was set at *p* < 0.05. GraphPad Prism 8 (GraphPad Software) and SPSS 26 (IBM SPSS Inc.) were used for all the analyses.

## Results

### Decrease of serum sHLA-G was strongly correlated with relapse of MM

Since the normalized FLC ratio (FLCr) is an indicator for estimating the duration of progression-free survival and overall survival of MM (Iwama et al. 2013; Alhaj Moustafa et al. 2015), it was routinely measured in our MM patients. According to an FLCr study on smoldering MM (Dispenzieri et al. 2008), abnormal FLCr (< 0.125 or > 8) was associated with a hazard ratio of 2.3 for symptomatic MM.

**Fig. 1:**
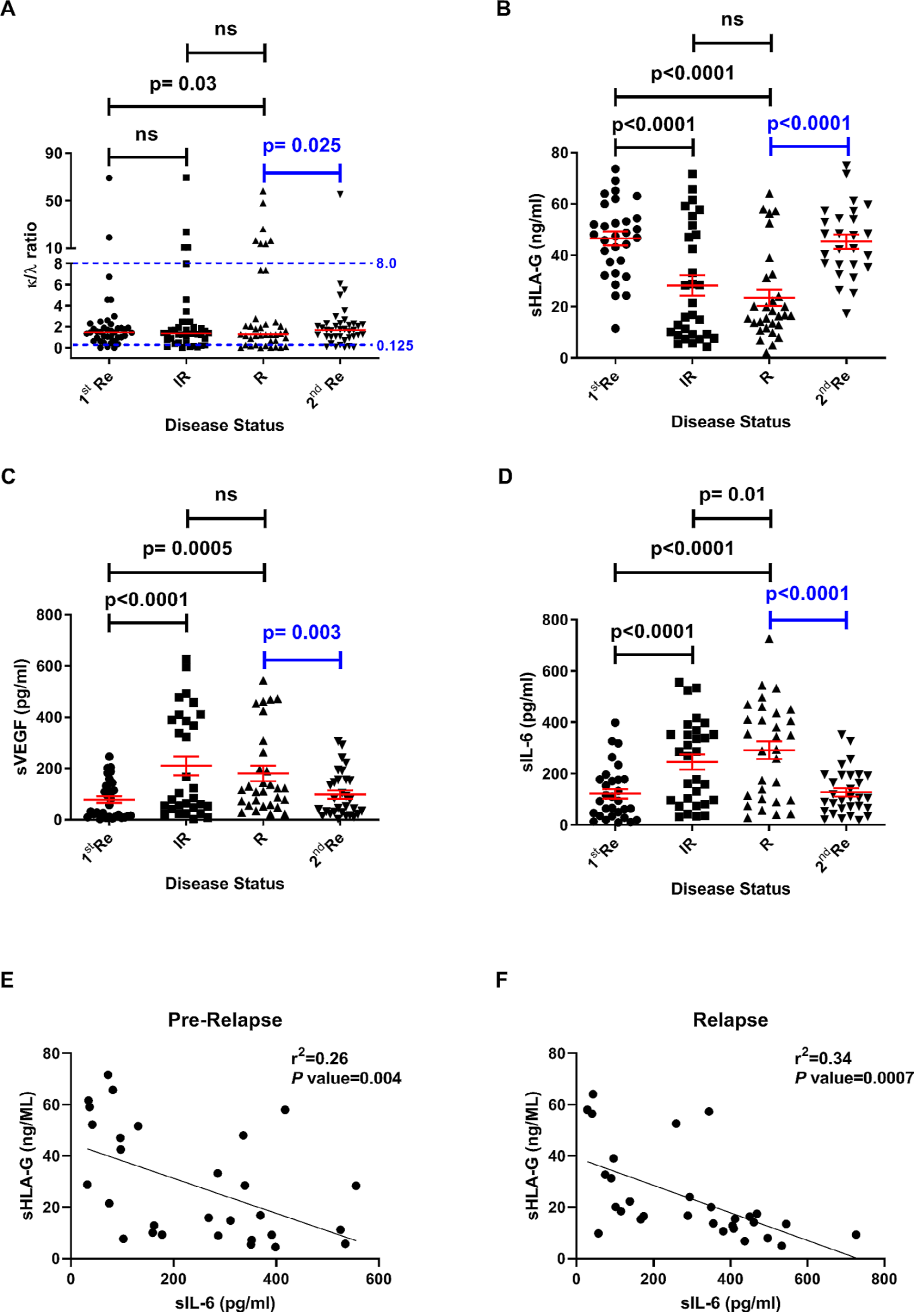
Dynamic changes of the FLCr, sHLA-G, VEGF, and IL-6 levels were shown in MM patients. Serum **A** FLCr, **B** sHLA-G, **C** VEGF, and **D** IL-6 levels were analyzed in different MM phases over time. Sera were collected on four phases as follows: the first remission (1st Re), imminent relapse (IR), relapse (R), and the second remission (2nd Re) after initial therapy. Short horizontal lines indicated median values in each group. The serum levels of IL-6 were reversely correlated with the levels of sHLA-G on **E** IR and **F** R phases using Pearson’s correlation coefficients. An abnormal FLCr was defined as a value < 0.125 or > 8. The differences of FLCr between two related groups were analyzed using McNemar test. Paired *t*-test was applied to assess the differences between two phases. Statistical significance was defined as *p* < 0.05. ns, not significant

We stratified our MM patients by FLCr, and measured their serum concentrations of sHLA-G, VEGF, and IL-6 during follow-up visits after the induction therapy. In Fig. 1A, there was more abnormal FLCr in relapse than in the first remission (35% vs. 13%, *p* = 0.03) or in the second remission (35% vs. 8%, *p* = 0.025). Figure 1A also showed that the sHLA-G levels of MM patients decreased significantly during IR (median level 19.2 ng/mL, *p* < 0.0001) and relapse (16.6 ng/mL, *p* < 0.0001), compared to the first remission (47.7 ng/mL). For patients in relapse, after a salvage therapy, their sHLA-G levels restored during the second remission (median 45.2 ng/mL), which was similar to their sHLA-G levels during the first remission. In contrast to the reduction of sHLA-G levels during IR and relapse phases, their serum levels of VEGF and IL-6 elevated remarkably compared to the levels during the first remission (VEGF: remission 44.9 pg/mL *versus* 119.7 pg/mL at IR [*p* < 0.0001] or *versus* 93.8 pg/mL at relapse [*p* = 0.0005]; IL-6: remission 93.0 pg/mL *versus* 277.0 pg/mL at IR [*p* < 0.0001] or *versus* 319.2 pg/mL at relapse [*p* < 0.0001])(Fig. 1B, C). When the patients achieved their second remission, these two cytokines decreased to the levels (65.8 pg/mL VRGF and 104.9 pg/mL IL-6) comparable to the levels at their first remission.

Moreover, linear regression analyses were performed to determine correlations between serum sHLA-G and IL-6 (Fig. 1D, *r*^2^ = 0.26, *p* = 0.004 for IR subjects; Fig. 1E, *r*^2^ = 0.34, *p* = 0.0007 for relapsed subjects). These results showed significant inverse correlations between sHLA-G and IL-6 in MM patients, but there was no correlation between sHLA-G and VEGF (data not shown). Because of the conspicuous changes of sHLA-G, VEGF, and IL-6 between different MM phases, we speculated that these factors could be relevant to angiogenesis, which impacted disease relapse. We thus explored the interplays among these factors in vitro.

**Fig. 2:**
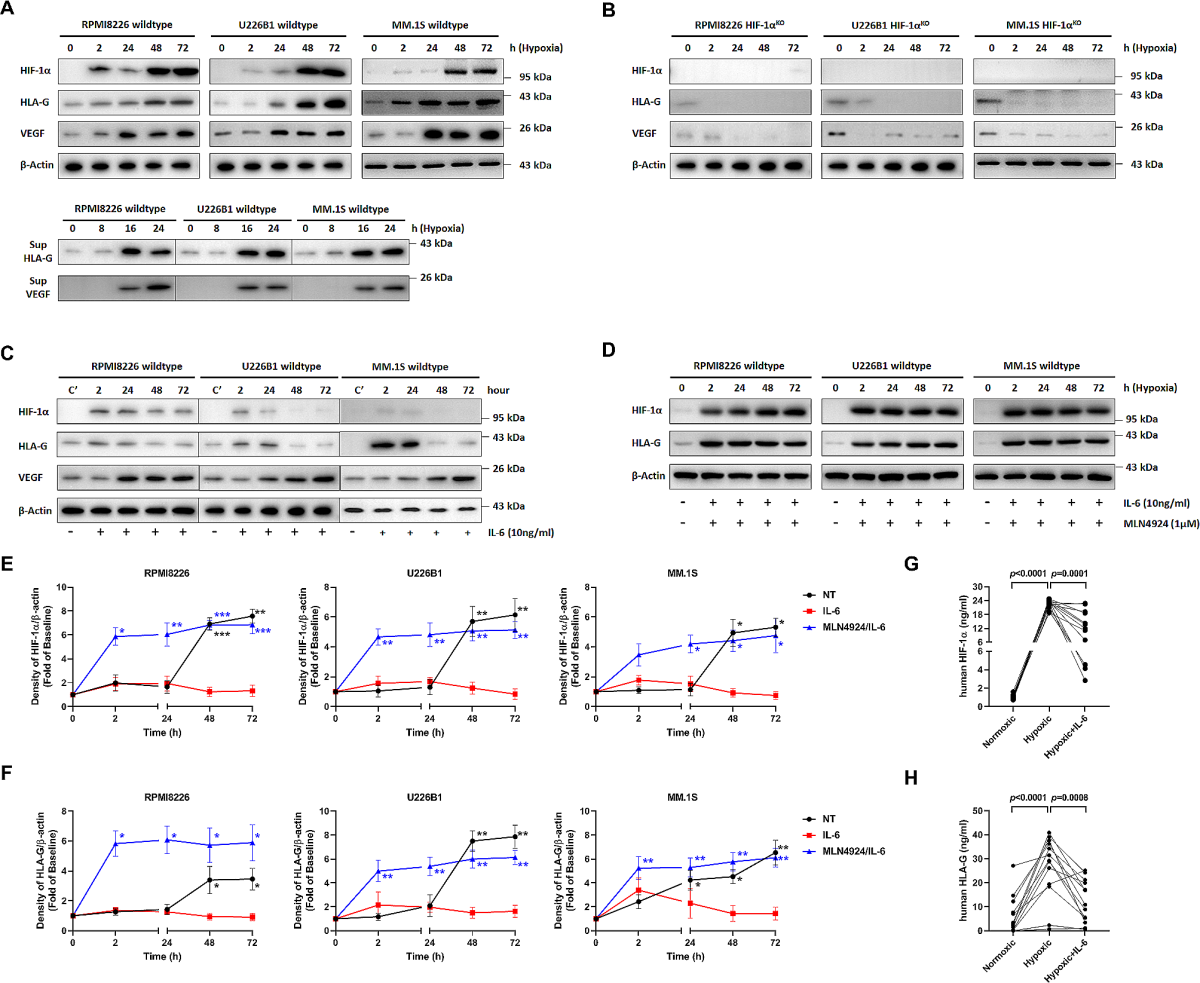
Hypoxia augmented the expression of HLA-G and VEGF associated with HIF-1α activity. Myeloma cells were incubated in a humidified atmosphere (normoxia) at time zero and changed to 3% O_2_ (hypoxia) over time. **A** Cells were harvested as the time indicated. Total cell lysates were analyzed by immunoblotting for HIF-1α, HLA-G, and VEGF. HLA-G and VEGF released in culture supernatants were also detected by Western blotting. **B.** To verify the role of HIF-1α in regulation of HLA-G and VEGF expressions, CRISP/Cas9 system was used to knock out *HIF-1α* (HIF-1α^KO^) in myeloma cells. **C.** Myeloma cells were treated with IL-6 (10 ng/ml) in 3% O_2_ over time. Whole cell extracts were separated by SDS-PAGE and analyzed by immunoblotting as the time indicated. **D.** MM cells treated with IL-6 over time were pretreated for 30 min with 1 µM MLN4924, a selective inhibitor of the NEDD8-Activating Enzyme (NAE). The densitometry data of HIF-1α (**E**) and HLA-G (**F**) from Western blot were normalized to that of β-actin. Protein levels at time zero were set as the baseline. Primary MM cells (*n* = 13) were cultured in medium with or without IL-6 under normoxia or hypoxia. The protein levels of HIF-1α (**G**) and HLA-G (**H**) were analyzed by ELISA. A paired *t*-test was applied to assess the differences between two groups. All experiments were performed in triplicate, and results were presented as the mean ± standard error of the mean. Asterisks represent significant differences from pairwise comparison between the non-treated cells (blue line), the cells treated with MLN4924 plus IL-6 (black line), and/or those treated with IL-6 (red line). *P < 0.05; **P < 0.01; ***P < 0.001. β-actin was used to quantify total proteins. Representative images were shown from one of three independent experiments. Sup, supernatant

### Hypoxia regulated the expression of HIF-1α and subsequent expression of HLA-G

Since the BM niche is physiologically hypoxic, we first examined the expression of HIF-1α, HLA-G, and VEGF in MM cells cultured in 3% of O_2_. The expression of HIF-1α, HLA-G, and VEGF in cultured MM cells increased significantly over time (Fig. 2A). Simultaneously, the secreted forms of HLA-G and VEGF also elevated markedly over time. To investigate whether HIF-1α controlled expressions of HLA-G and VEGF, *HIF-1α* knockout was established by CRISPR/Cas9 in MM cells. As shown in Fig. 2B, substantial down-regulation of HLA-G and VEGF was concordantly found in the three MM cell lines when HIF-1α protein expression was abolished. We next investigated the direct impacts of IL-6 (10 ng/mL) on mRNA and protein levels of HIF-1α and HLA-G in IL-6 treated MM cells by qPCR and immunoblot. No remarkable changes of *HIF-1α* and *HLA-G* transcripts were found (Figure S3). In contrast, protein expressions of HIF-1α and HLA-G in the three types of MM cells almost vanished after 48-h treatment with IL-6 (Fig. 2C). Additionally, the expression of VEGF was preserved after the treatment with IL-6. Since IL-6 reduced the protein levels of both HIF-1α and HLA-G without altering their mRNA expression, we next examined whether a post-translational mechanism, such as ubiquitination-mediated degradation, was involved. HIF-1α has been known as a substrate of the cullin subunits of Cullin-RING-ligases (CRLs), the largest family of multiunit E3 ubiquitin ligases, for protein degradation (Curtis et al. 2015; Lin et al. 2021). This cullin neddylation can be impaired by targeting NEDD8 (neural precursor cell expressed developmentally downregulated protein 8), which is a ubiquitin-like molecule that activates CLRs (Soucy et al. 2009). Thus, we employed NEDD8-activating enzyme inhibitor MLN4924 to explore whether this machinery of protein degradation of HIF-1α and HLA-G occurred in MM cells. Indeed, IL-6-induced decrease of HIF-1α and HLA-G proteins was negated by the co-treatment with MLN4924 in all three cell lines (Fig. 2D). Inactivation of CRLs by MLN4924 significantly stabilizes cellular levels of HIF-1α and HLA-G. Figure 2E-F showed the averaged changes of protein densities under differential treatments over time from three independent experiments.

HIF-1α could mediate expressions of HLA-G and VEGF in MM cells under hypoxia. IL-6 promoted degradation of HIF-1α and HLA-G proteins likely via the ubiquitin pathway. Similar to the MM cell lines, the protein expressions of HIF-1α (Fig. 2G) and HLA-G (Fig. 2H) in primary MM cells from 13 patients also showed similar responses to hypoxia and IL-6. HIF-1α and secreted HLA-G from the primary MM cells were significantly elevated in the hypoxic condition compared to the normoxic condition. IL-6 also substantially attenuated expressions of these two proteins in the primary cells under hypoxia. To validate the in vitro observation, we next established two mouse models of human MM by orthotopic engraftments of RPMI8226 and IL-6-expressing RPMI8226 cells into the immunodeficient mice (ASID mice).

### Animal model of human MM

**Fig. 3:**
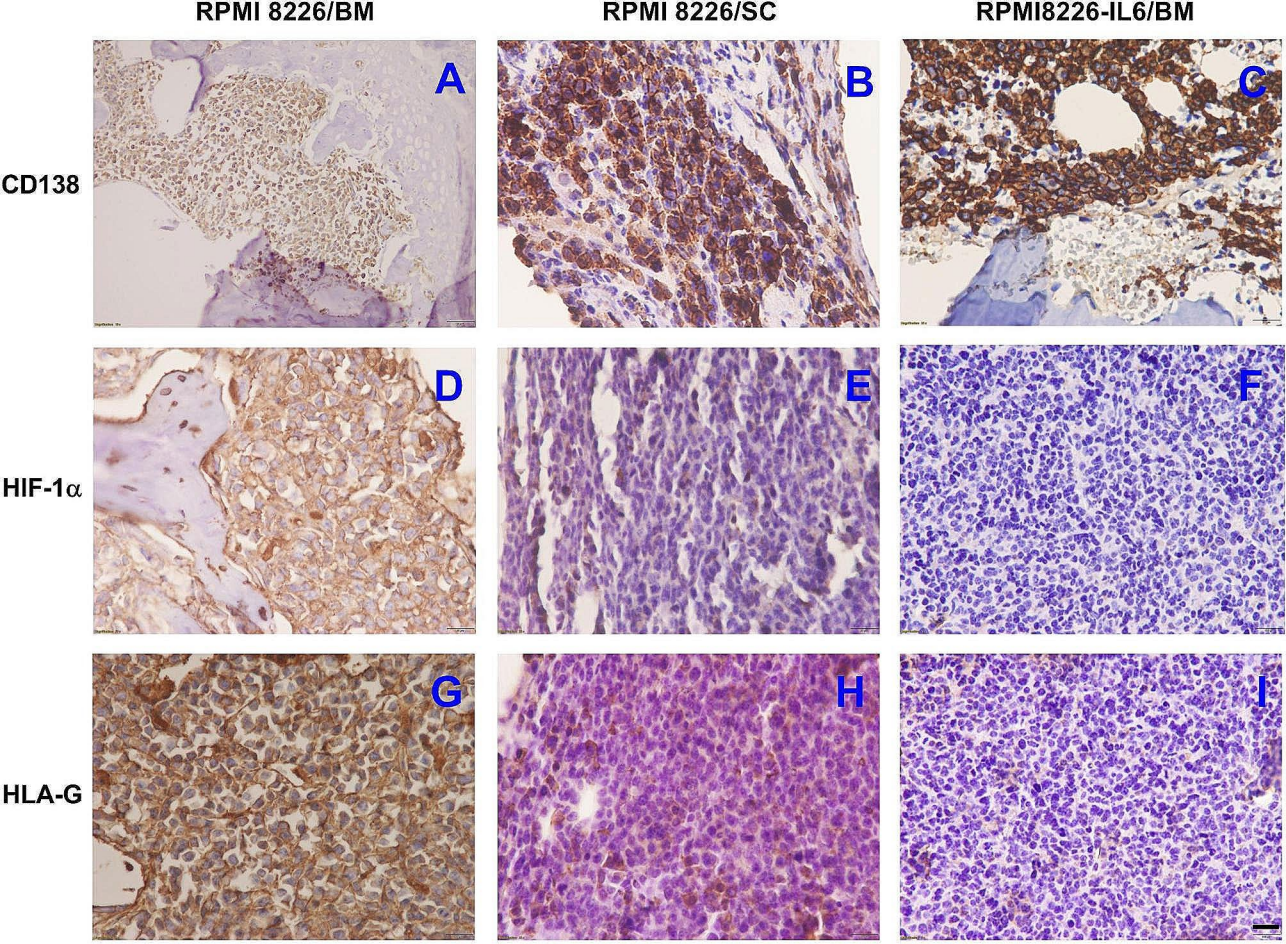
Immunohistochemical analyses of human myeloma cells expressing CD138 (a nonmalignant/malignant plasma cell marker), HIF-1α, and HLA-G in xenografted ASID mouse models. The tibia BM cavities of immunodeficient mice were engrafted with RPMI8226 cells (**A**, **D**, **G**) or IL-6 expressing RPMI8226 cells (**C**, **F**, **I**) by direct intratibial injection. To compare with the extremely hypoxic BM environment, a hollow tibia filled with RPMI8226 cells was subcutaneously implanted in ASID mice (**B**, **E**, **H**). The grafted tissues were analyzed by immunohistochemistry 35 days later post-transplantation. The representative images (×500 magnification; scale bar, 20 μm) showed MM cells were stained with antibodies specific for human CD138 (**A**, **B**, **C**), HIF-1α (**D**, **E**, **F**), or HLA-G (**G**, **H**, **I**), and positive antibody stain was shown in dark brown color. The experiments were performed in two mice per group

To test whether the effects of hypoxia and IL-6 on HIF-1α and HLA-G expression could be recapitulated in the BM environment in vivo, we performed direct intratibial injection with RPMI8226 or with IL-6-expressing RPMI8226 cells into the ASID mice, and observed expressions of HIF-1α and HLA-G after 35 days of the engraftments. Figure 3F and I showed that IL-6-expressing RPMI8226 cells significantly lowered expressions of HIF-1α and HLA-G in the hypoxic BM of the ASID mice, compared to the engraftment of only parental RPMI8226 cells. Further, to compare the effects of oxygen levels, we filled a hollow tibia with RPMI8226 cells and implanted this RPMI8226-packed tibia into the subcutaneous area of the ASID mice. The grafted bone sections were analyzed by immunohistochemistry (Fig. 3). The expressions of HIF-1α and HLA-G in RPMI8226 cells were substantially higher in the extremely hypoxic BM (Fig. 3D and G) than in the more oxygenated tibia implanted in the subcutaneous region (Fig. 3E and H). Taken together, these in vivo findings indeed were consistent with those found in vitro.

### IL-6 augmented degradation of HIF-1α and HLA-G via PARKIN

**Fig. 4:**
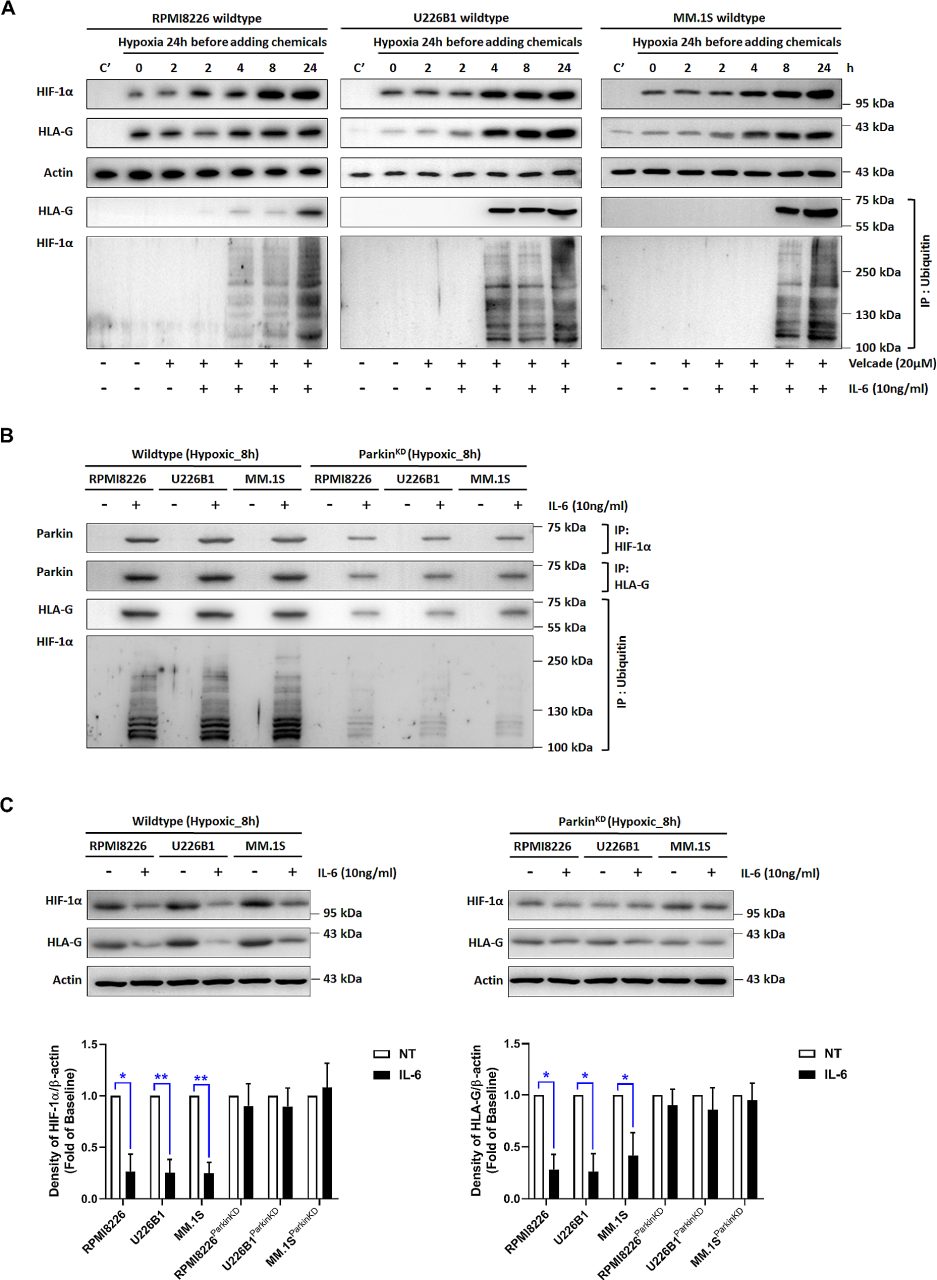
IL-6 attenuated expressions of HIF-1α and HLA-G through ubiquitination. **A** RPMI8226 (left panel), U226B1 (middle panel), and MM.1s (right panel) cells were subjected to treatments with IL-6 and bortezomib (Velcade) under a hypoxic condition. Cells were cultured in normoxia as controls (C’). β-actin was used to quantify total proteins. Immunoprecipitants using anti-ubiquitin were separated by SDS-PAGE, and analyzed by Western blot using HIF-1α-specific and HLA-G-specific antibodies. **B** Ubiquitination of HIF-1α and HLA-G in IL-6-treated MM cells was associated with the PARKIN activity. Knockdown of *PARKIN* at 24-h post-transfection was verified by real-time PCR (data not shown). The proteins co-precipitated with HIF-1α or HLA-G were also analyzed by Western blot using a PARKIN-specific antibody. **C** Densitometry data of HIF-1α and HLA-G were normalized to β-actin. MM cells were incubated in hypoxia for 8 h. Representative images were shown from one of the three independent experiments. Statistical analysis using pairwise comparison was employed to distinguish protein levels between two groups. *P < 0.05; **P < 0.01. These cell-based studies were performed at least three times, and showed similar findings. IP, immunoprecipitation

The NEDD8 associated CLRs have been known to control degradation of about 20% of proteasome-regulated proteins (Petroski and Deshaies 2005; Deshaies and Joazeiro 2009). Since MLN4924 regulated proteolytic processing of HIF-1α and HLA-G (Fig. 2), we further used proteasome inhibitor bortezomib (Velcade) to verify their protein down-regulation through the ubiquitination machinery. We immunoprecipitated ubiquitinated proteins, and performed Western blot analysis for HIF-1α and HLA-G. The ubiquitinated HIF-1α and HLA-G proteins were identified in the three myeloma cell lines after 4-h treatment with IL-6 (Fig. 4A). It is known that PARKIN, an E3-ubiquitin ligase(Gladkova et al. 2018), could be up-regulated at the transcription level by IL-6(Tyrrell et al. 2020). We thus explored whether co-immunoprecipitation of PARKIN with HIF-1α and HLA-G from myeloma cells could be expedited by IL-6. As shown in Fig. 4B, the expression of PARKIN in the MM cell lines was enhanced only in the presence of IL-6. Knockdown of *PARKIN* reduced the activity of PARKIN and ubiquitination of HIF-1α and HLA-G. In parallel, reduction of HIF-1α and HLA-G protein expressions by IL-6 was counteracted by knockdown of *PARKIN* in the MM cells (Fig. 4C). Collectively, these findings revealed that protein degradation of HIF-1α and HLA-G was enhanced by IL-6 via the PARKIN activity.

### sHLA-G inhibited capillary tube formation

**Fig. 5:**
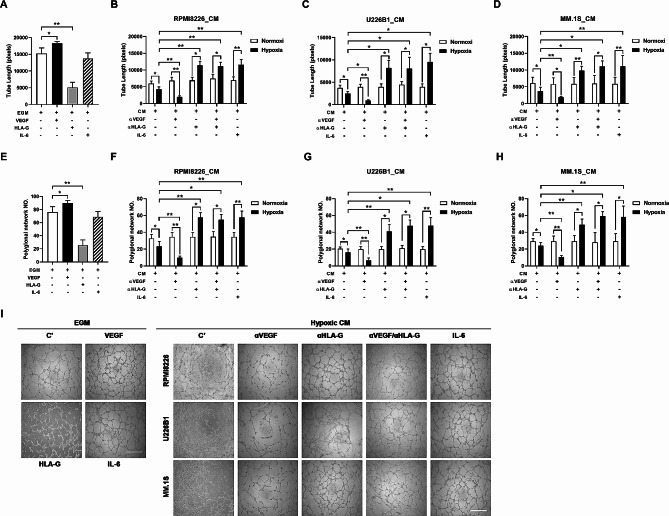
sHLA-G, VEGF, and IL-6 were involved in tube formation of HUVEC in vitro. The quantitative analysis showed that cumulative tube length (**A-D**) and polygonal network number (**E-H**) in various culture conditions of HUVEC. To investigate the effects of sHLA-G and IL-6 in the tube formation, sHLA-G (1 µg/mL) and IL-6 (10 ng/mL) were added to EGM containing 2% of FBS for HUVEC culture (**A** and **E**). **I** Representative phase-contrast images of the tube formation of HUVEC in EGM (left panel) and CM (right panel), which were the media collected from myeloma cell culture under hypoxia. The bar graph showed mean total tube length obtained using the Image-Pro Plus software. Cell interconnecting networks were counted as polygonal nodes. CM was collected from culture media of the MM cell lines, such as RPMI8226, U266, and MM.1s, after 16 h of cell culture in 21% or 3% of oxygen. To explore the effects of IL-6 on VEGF and sHLA-G, IL-6 (10 ng/mL) was added to the MM cell culture first. Anti-HLA-G or anti-VEGF neutralizing antibodies were used to eliminate secreted HLA-G or VEGF in the CM, which would be used for *in-vitro* angiogenesis assays. The error bars indicate the SEM of at least three independent experiments. Scale bar: 100 mm. *p < 0.05, **p < 0.01 (Student’s *t*-test). Representative images were shown from one of three independent experiments

**Fig. 6:**
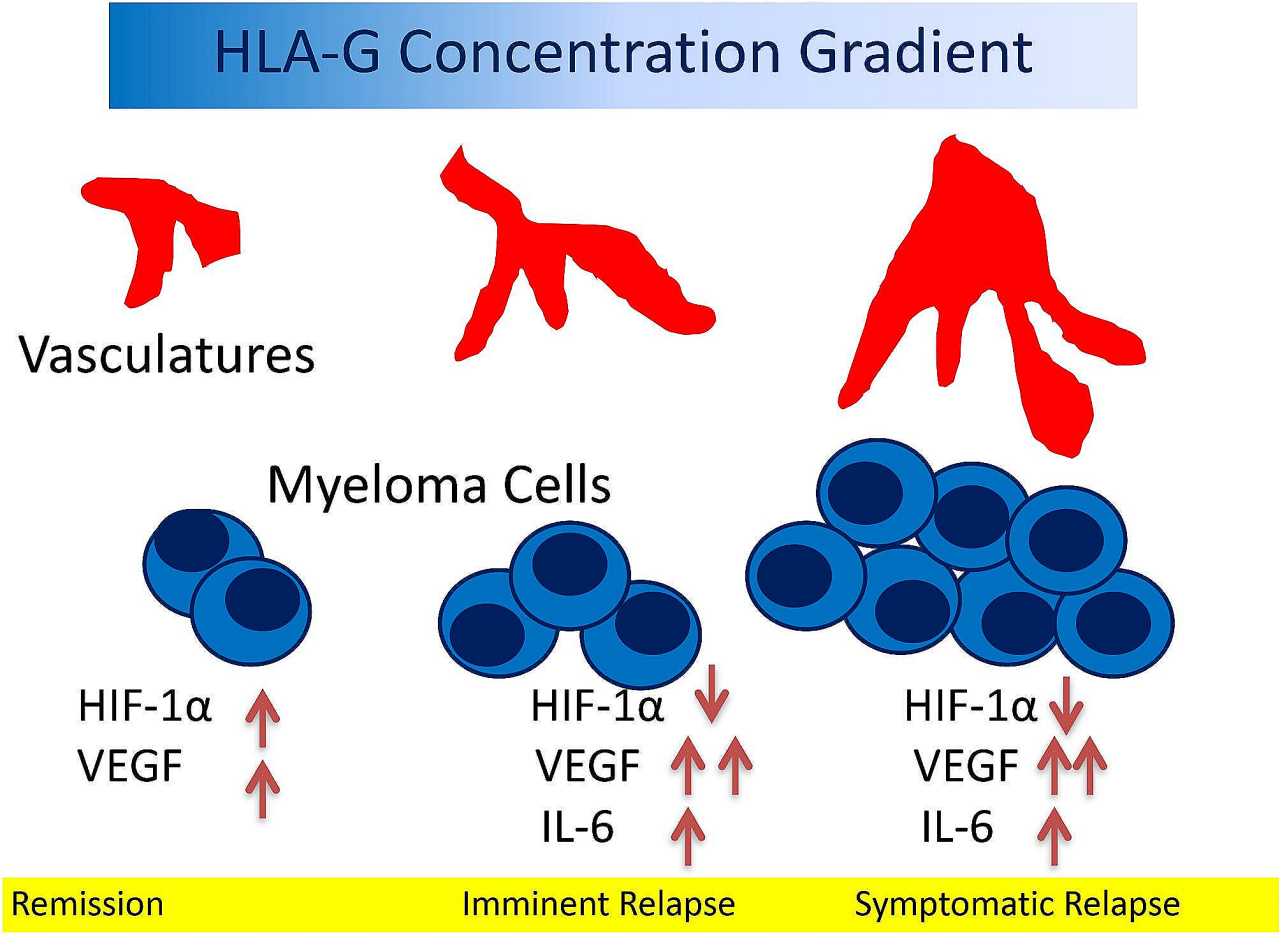
A schematic summary. The decline of serum HLA-G is tied to IL-6 expression and HIF-1α degradation. IL-6 enhances expansion of myeloma cells and consequently increases VEGF and myeloma burden. The simultaneous reduction of HLA-G turns on the angiogenesis switch. These *in-vitro* data may explain the functions of HLA-G during MM progression

To investigate the interactions among sHLA-G, VEGF, and IL-6 in angiogenesis, HUVEC was then cultured in sHLA-G and sHLA-G-depleted conditioned media. The tube structures mimicking angiogenesis in vitro were quantitated by measuring the cumulative tube length (Fig. 5A-D) and the number of the polygonal network (Fig. 5E-H) are shown. Representative phase-contrast images of the tube formation of HUVEC in EGM (left panel) and CM (right panel) were shown in Fig. 5I. As shown in Fig. 4A and E, the treatment with sHLA-G significantly inhibited capillary tube formation of HUVEC that were cultured in a growth factor-reduced Matrigel under normoxia. Conversely, tube formation was remarkably enhanced following VEGF treatments. The increase of tube formation however was not found after adding IL-6 (Fig. 5A, E). Tube formation was inhibited when HUVEC was incubated in hypoxic CM, and not in normoxic CM. As HLA-G and VEGF were secreted in the CM collected from MM cells cultured under hypoxia (Fig. 2A), HLA-G and VEGF were individually depleted in the CM using neutralized antibodies, and HLA-G-depleted and/or VEGF-depleted CM were used to culture HUVEC for *in-vitro* angiogenesis studies. Depletion of HLA-G in this CM collected from hypoxic MM culture enhanced tube formation of HUVEC, so did an add-on treatment with IL-6. In contrast, depletion of VEGF in the CM collected from hypoxic MM culture inhibited tube formation of HUVEC. Interestingly, elimination of both HLA-G and VEGF in the CM collected from hypoxic MM culture resulted in similar degrees of tube formation of HUVEC as elimination of HLA-G alone. However, treatments with anti-VEGF antibody, anti-HLA-G antibody, or IL-6 in the CM collected from normoxic MM culture did not influence the tube formation of HUVEC. Taken together, HLA-G played a crucial role in inhibition of angiogenesis, and IL-6 could counteract this inhibitory effect of HLA-G through ubiquitin-mediated protein degradation (Fig. 4). In summary, Fig. 6 depicts the interaction among these factors and myeloma cells in promoting angiogenesis.

## Discussion

MM remains an incurable malignancy as most patients inevitably experience relapse. Angiogenesis in BM, a hallmark of MM progression, is stimulated by angiogenic cytokines generated by myeloma cells and BMM components. In this study, we identified a new function of sHLA-G in governing angiogenesis. High levels of sHLA-G were constantly expressed in MM patients on remission after given an induction therapy. Their high levels of sHLA-G sharply declined when the patients developed relapse. This early warning sign could even be found three months before recurrence. The levels of VEGF and IL-6 also elevated before MM progressed. Notably, similar to the findings by Leleu et al., the association between overall survival and sHLA-G levels in our MM patients was also not significant (Leleu et al. 2005). Mechanistically, IL-6 down-modulated sHLA-G by activating PARKIN to degrade HIF-1α and HLA-G. The intact expression of VEGF was attributed to IL-6 effect along with STAT3 activity(Cohen et al. 1996), albeit HIF-1α, the major modulator for VEGF expression, was degraded. Additionally, by mimicking the condition of BM under physiological hypoxia, which was consistent with the findings in mouse BM (Fig. 3), we found that HIF-1α mediated expressions of HLA-G and VEGF. In our *HIF-1αKO* myeloma cells, HIF-2α expression under hypoxia was not affected by knockout of *HIF-1α*. Although HIF-1 and HIF-2 overlap in function, HIF-1α and HIF-2α show different specificity for their individual transcriptional targets (Albadari et al. 2019). From in vitro angiogenesis assays, HLA-G curtailed tube formation of HUVEC, and HLA-G-depleted CM collected from hypoxic MM culture restored vascularization. Hence, increase of IL-6 production could suppress HLA-G expression and initiate angiogenesis. From this *in-vitro* study, we found that HLA-G could inhibit angiogenesis. Importantly, the dynamic changes of sHLA-G in cell culture experiments as well as in MM patients at different phases suggest that the level of sHLA-G is sensitive to progression status of MM. Whether this could be developed into a useful clinical prognostic tool remains to be determined, as one of the limitations in this study is that the patients were recruited from a single medical center in Taiwan and were all of homogeneous ethnicity (East Asian origin). In the future, it is necessary to examine MM patients of different ethnicities to verify the impacts of various sHLA-G levels on the prognosis of ethnically-diverse patients with MM.

The expression of HLA-G has been demonstrated to be enhanced through HIF-1 under hypoxia and hypoxia-simulating conditions in the tumor microenvironment (Mouillot et al. 2007; Garziera et al. 2017). In agreement with these reports, our data probably showed that HIF-1α was associated with HLA-G expression in MM cells under hypoxia. Additionally, IL-6 diminished expressions of HLA-G and HIF-1α through PARKIN-mediated ubiquitination. IL-6 has been found to up-regulate PARKIN expression (Tyrrell et al. 2020), and PARKIN could target HIF-1α for ubiquitination and degradation (Liu et al. 2017). We observed ubiquitinated HIF-1α and HLA-G formation within 8 h after a treatment with IL-6 (Fig. 4A). A genetic inhibitor of *PARKIN* abolished ubiquitination of HIF-1α and HLA-G (Fig. 4B).

In line with the findings by Fons et al. (Fons et al. 2006), sHLA-G1 inhibits endothelial cell tube formation and induces apoptosis through engagement of CD160. The treatment with sHLA-G diminished formation of tube networks in endothelial cells. This observation agreed with that HUVEC incubated in sHLA-G containing conditioned medium collected from MM cell culture yielded less tube formation (Fig. 5). Also, as shown in the experiment using the immunodeficiency mouse model (Fig. 3), we found that HLA-G expression could be modulated by hypoxia and by IL-6 production. On the other hand, these mechanistic findings from the in vitro experiments and xenograft models may not completely mimic situations in the human bone marrow milieu (Walsh et al. 2017). For verification, future direction should include development of a humanized mouse model for mechanistic study of the neovascular formation in MM.

Currently, numerous immune-based therapeutics for relapsed/refractory MM show promising anti-cancer effects (Minnie and Hill 2020). These therapies include chimeric antigen receptor (CAR) T cells, bispecific antibodies mediating T-cell recruitment, and immune checkpoint inhibition. However, reports on the overall responses and the responses of durable functional anti-myeloma T cells are limited. Immunosuppression of the microenvironment and tumor loads are two crucial parameters to be considered. HLA-G prominently play a role to suppress a broad spectrum of immune effectors (Rouas-Freiss et al. 2014). In our investigation, a low level of sHLA-G was found on the IR phase (3 months before symptomatic relapse). We also noticed low levels of serum FLC in our patients, which could be associated with tumor load (Bradwell et al. 2003; Katzmann et al. 2005). This study did not probe into the prolonged consequences of sHLA-G levels, though it is conceivable that long-term changing expression of sHLA-G in patients with MM could be associated with their immunosuppressive status. HLA-G interacts with immunoglobulin-like transcript 2 and 4 receptors (ILT2 and ILT4), which consequently could inhibit cytotoxic T cells, natural killer cells, and B cells, induce T cell anergy, modulate myeloid cells, and promote regulatory T cell activities (Carosella et al. 2021). As the aim of this study was not to identify the prolonged and complex impacts of sHLA-G on the immune presentation of the patients with MM, these should be investigated in the future for potential clinical and therapeutic applications.

In conclusion, inducing HLA-G degradation through IL-6/PARKIN activity, which enhances ubiquitination of HIF-1α and HLA-G, strengthens neovascular tube formation particularly under hypoxia. To prevent relapse of cancer, particularly angiogenesis, remains the critical therapeutic target. Thus, it is valuable to develop maintenance therapy with IL-6 inhibitors or ubiquitination blockades to prevent progression of MM. Further, it is worthwhile to investigate the phenomenon of sHLA-G reduction as a warning biomarker of disease progression in the future.

### Electronic supplementary material

Below is the link to the electronic supplementary material.

Supplementary Material 1

## Data Availability

The datasets used and/or analyzed during the current study available from the corresponding author on reasonable request.

## Abbreviations

AUC: Area under ROC curve
BM: Bone marrow
BMM: BM microenvironment
CRP: C-reactive protein
FLCr: Free light-chain ratio
HIF: Hypoxia-inducible factor
HLA-G: Human leukocyte antigen G
HUVEC: Human umbilical vein endothelial cell
IL-6: Interleukin 6
ILT: Immunoglobulin-like transcript
IR: Imminent relapse
LDH: Lactate dehydrogenase
MM: Multiple myeloma
OR: Odds ratio
ROC: Receiver operating characteristic
VEGF: Vascular endothelium growth factor
