# Supplementary material for: The implication of serum HLA-G in angiogenesis of multiple myeloma
Source: Mol Med. 2024 Jun 14;30:86. doi: 10.1186/s10020-024-00860-5 (PMC11177474; doi:10.1186/s10020-024-00860-5)

**Supplementary Information**

**The Implication of Serum HLA-G in Angiogenesis of Multiple Myeloma**

Chi Wang^1^, Nai-Wen Su^2,3^, Kate Hsu^4,5^, Chen-Wei Kao^6^, Ming-Chih Chang^2,7^, Yi-Fang Chan^2,6^, Ken-Hong Lim^2,6,7^, Yi-Hao Chiang^2,6,7^, Yu-Cheng Chang^2,6,7^, Meng-Ta Sung ^2^, Hsueh-Hsia Wu^8^, Caleb G. Chen^*2,3,6,8^

Table S1. Demographics of the recruited patients with multiple myeloma

Figure S1. Effects of IL-6 in regulating HIF-1α and HLA-G expression.

Figure S2. The expression of HIF-1α and HIF-2α of *HIF-1αKO* myeloma cells incubated under normoxic condition or 3% O_2_ for 24 h.

Figure S3. Effects of IL-6 in regulating HIF-1α and HLA-G expression.

Supplementary Table 1. Demographics of the recruited patients with multiple myeloma

| Variable | All Patients (n=57) |
| --- | --- |
| Age, median (range), years | 67 (50-89) |
| Sex |  |
| Male | 30 (52%) |
| Female | 27 (48%) |
| Stage at diagnosis (ISS) |  |
| I | 6 (11%) |
| II | 12 (21%) |
| III | 39 (68%) |
| Immunoglobulin heavy chain |  |
| IgG | 39 (68%) |
| IgA | 12 (21%) |
| Light chain only | 6 (11%) |
| Involved light chain |  |
| Kappa | 31 (55%) |
| Lambda | 26 (45%) |
| Outcome |  |
| CR | 31 (55%) |
| VGPR | 8 (14%) |
| PR | 18 (31%) |
| eGFR (ml/min/1.73m2) |  |
| ≥60 | 32 (57%) |
| <60 | 25 (43%) |

Supplementary Figure 1.

**The expression of HIF-1α and HLA-G of myeloma cells incubated under different O_2_ concentration.**

Myeloma cells were incubated in a humidified atmosphere (normoxia, C') at time zero and changed to 1-5% O_2_ (hypoxia) for 24 h. Cells were harvested and total cell lysates were analyzed by immunoblotting for HIF-1α, HLA-G. β-actin was used to quantify total proteins.


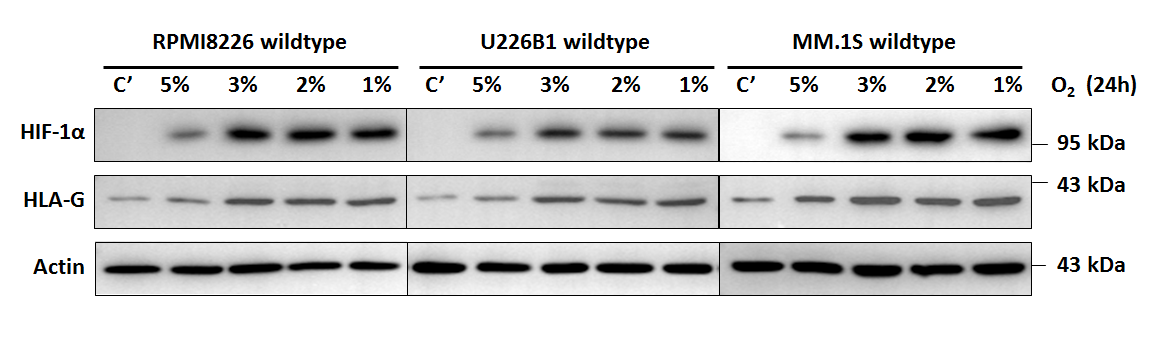


Supplementary Figure 2

**The expression of HIF-1α and HIF-2α of *HIF-1αKO* myeloma cells incubated under normoxic condition or 3% O_2_ for 24 h.** Cells were harvested and total cell lysates were analyzed by immunoblotting for HIF-1α, HIF-2α, and HLA-G. β-actin was used to quantify total proteins.


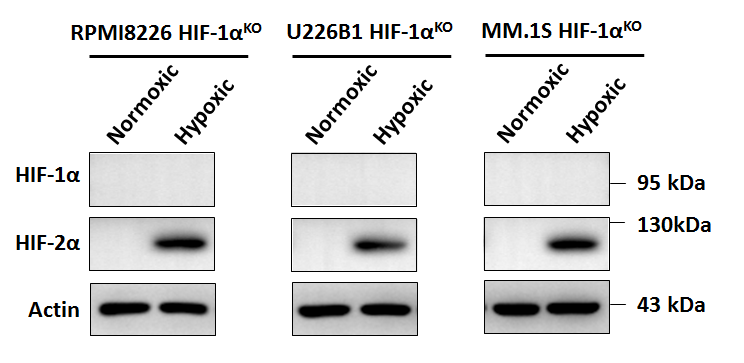


Supplementary Fig. 3

**Effects of IL-6 in regulating HIF-1α and HLA-G expression.**

Total cellular RNA was extracted at various time points as indicated using the RNeasy Mini Kit (Qiagen), and reverse-transcribed to cDNA using the High Capacity cDNA Reverse Transcription Kit (Applied Biosystems). Quantitative PCR (qPCR) was performed using the CFX Connect^TM^ Real-Time System and software (Bio-Rad). Products were detected using SYBR^®^ Green Master Mix. PCR primer sequences are listed as follows: HIF-1α Forward: 5’- CATAAAGTCTGCAACATGGAAGGT-3’

HIF-1α Reverse: 5’- ATTTGATGGGTGAGGAATGGGTT-3’

HLA-G Forward: 5’-TTGGGAAGAGGAGACACGGAACA-3’

HLA-G Reverse: 5’-AGGTCGCAGCCAATCATCCAC-3’. The human *RPL19* gene was used as an internal reference control for normalization.

A. Transcript level changes of HIF-1α after treatment with IL-6 (10ng/mL) in three MM cell lines.

B. Transcript level changes of HLA-G after treatment with IL-6 (10ng/mL) in three MM cell lines.

NT, non-treated


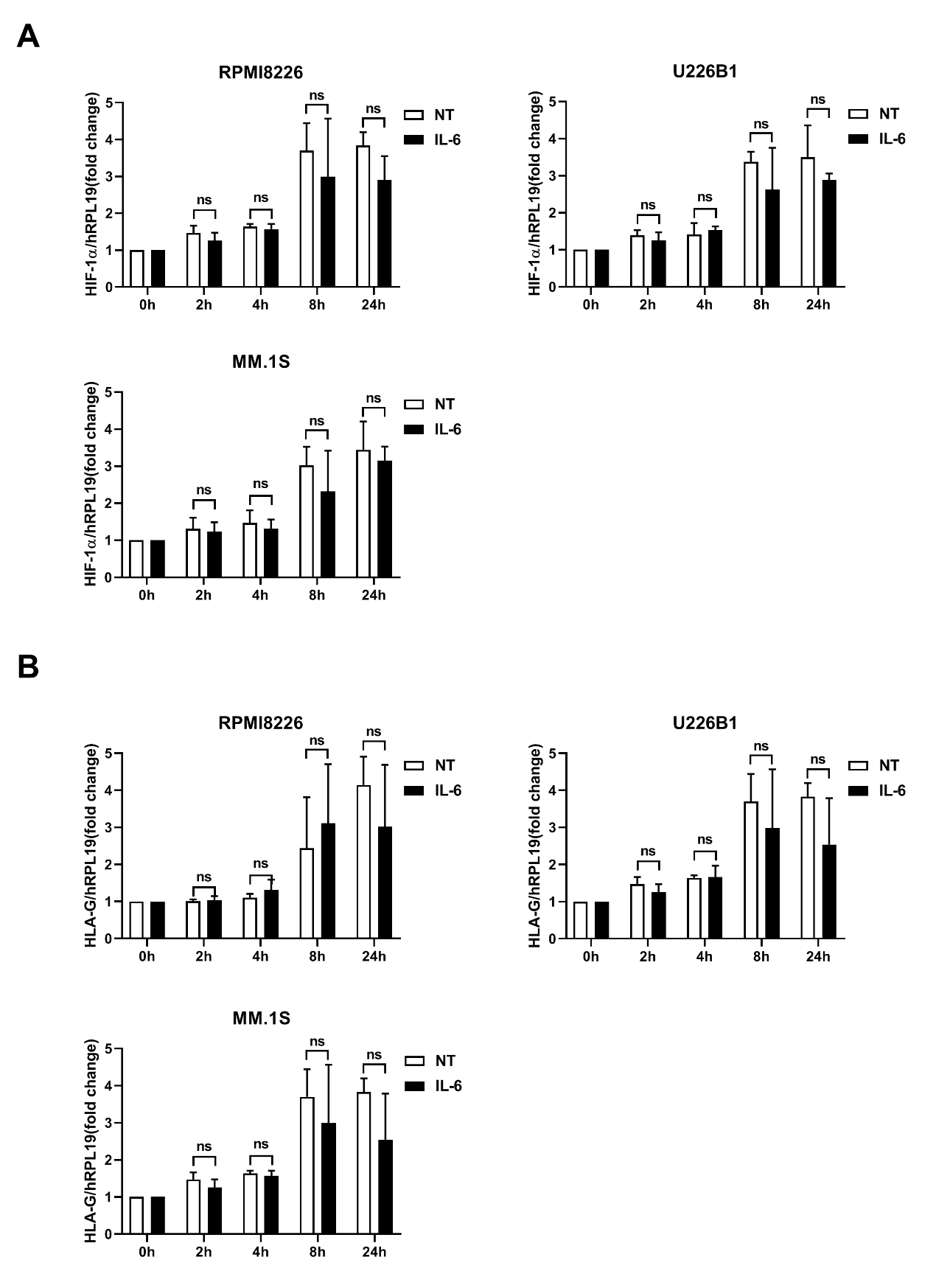

Supplement: Supplementary file 1 — Supplementary Material 1 [file 10020_2024_860_MOESM1_ESM.docx]
